# It Comes and Goes: Pediatric Chronic Spontaneous Urticaria

**DOI:** 10.7759/cureus.40006

**Published:** 2023-06-05

**Authors:** Ming Lee Chin

**Affiliations:** 1 Department of Paediatrics, Taiping Hospital, Perak, MYS

**Keywords:** omalizumab, antihistamine, pediatric dermatology, chronic spontaneous urticaria, chronic urticaria

## Abstract

Chronic spontaneous urticaria (CSU) is an underrecognized and underreported condition, even more so in the pediatric population. Due to its fugacious nature, the period between the onset of symptoms and the diagnosis of CSU is often long.

We discuss the case of a 10-year-old child who presented with a six-month history of recurrent, pruritic rash. Medical advice was sought on multiple occasions; however, no treatment was initiated. This resulted in the child and caretakers becoming increasingly worried. The child was subsequently diagnosed with CSU. Daily second-generation antihistamine was started, and the child responded well with marked improvement of symptoms.

Our case raises a pertinent point. It is crucial for physicians to be able to recognize and treat CSU according to evidence-based guidelines, as this condition may not only negatively affect the child’s quality of life, but its impact also extends to the caretakers.

## Introduction

Chronic spontaneous urticaria (CSU) is an underrecognized condition that can compromise quality of life. It typically takes a long time from the onset of symptoms to a proper diagnosis and correct treatment [1]. According to studies, the average time between the beginning of symptoms and diagnosis is 24 months [2].

The diagnosis of CSU in children is primarily based on clinical history and physical examination [1]. In addition, the physician should aim to identify any triggering factors, as well as rule out any sinister, systemic illnesses.

The 2021 international European Academy of Allergy and Clinical Immunology (EAACI)/Global Allergy and Asthma European Network (GA²LEN)/EuroGuiDerm/Asia Pacific Association of Allergy, Asthma and Clinical Immunology (APAAACI) guideline is the most widely used and accepted in terms of diagnosis as well as management of CSU in adults [3]. However, there are few guidelines targeted specifically at the pediatric population, mostly due to the lack of high-quality research to support therapeutic options in children [4]. Therefore, the management of pediatric CSU is generally extrapolated from studies involving adults. Second-generation antihistamines (sgAHs) remain the mainstay of treatment due to their safety profile [5].

We describe the case of a child who presented with a six-month history of recurrent pruritic skin rashes, which was later diagnosed to be CSU.

Due to the prolonged course of illness, CSU may not only impact the child’s quality of life but can also significantly affect the caregivers and parents as well due to missed time from work and feelings of anxiety [1,6]. This article aims at providing an overview of this potentially debilitating condition to assist physicians in better identifying and managing such patients.

## Case presentation

A 10-year-old girl was referred from a local healthcare facility for recurrent skin rashes for the past six months. On further history, the rash would appear in different areas of her body on a daily basis, regardless of time, and with no obvious triggering factors. More often than not, it would become generalized, involving the entire body. It was also pruritic in nature. There was no involvement or swelling of mucous membranes. The rash would usually resolve spontaneously within an hour of its initial appearance, with no residual lesions.

Otherwise, the child did not report any constitutional symptoms, such as loss of weight or appetite. She also denied any intercurrent illnesses, joint pains, alopecia, or oral ulcers. Her past medical history was unremarkable for any food or drug allergies. The child was the second of three siblings, and her parents were non-consanguineous. Apart from her younger brother who had well-controlled bronchial asthma, there were no family members with a similar rash, history of atopies, or systemic illnesses.

The child’s parents brought her to seek medical attention on multiple occasions in the span of six months, but she was not prescribed any treatment. Instead, the family was offered reassurance that it was a harmless allergic reaction. However, as the rash continued to wax and wane on a daily basis, they grew increasingly anxious.

**Figure 1:**
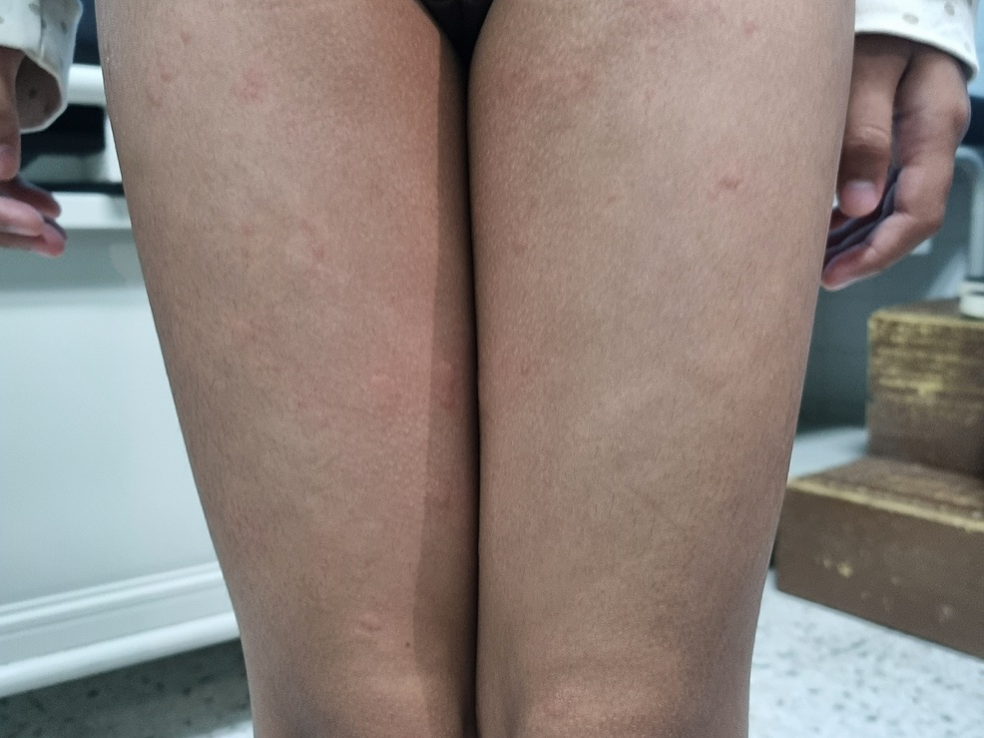
Urticaria over the child’s bilateral lower limbs.

**Figure 2:**
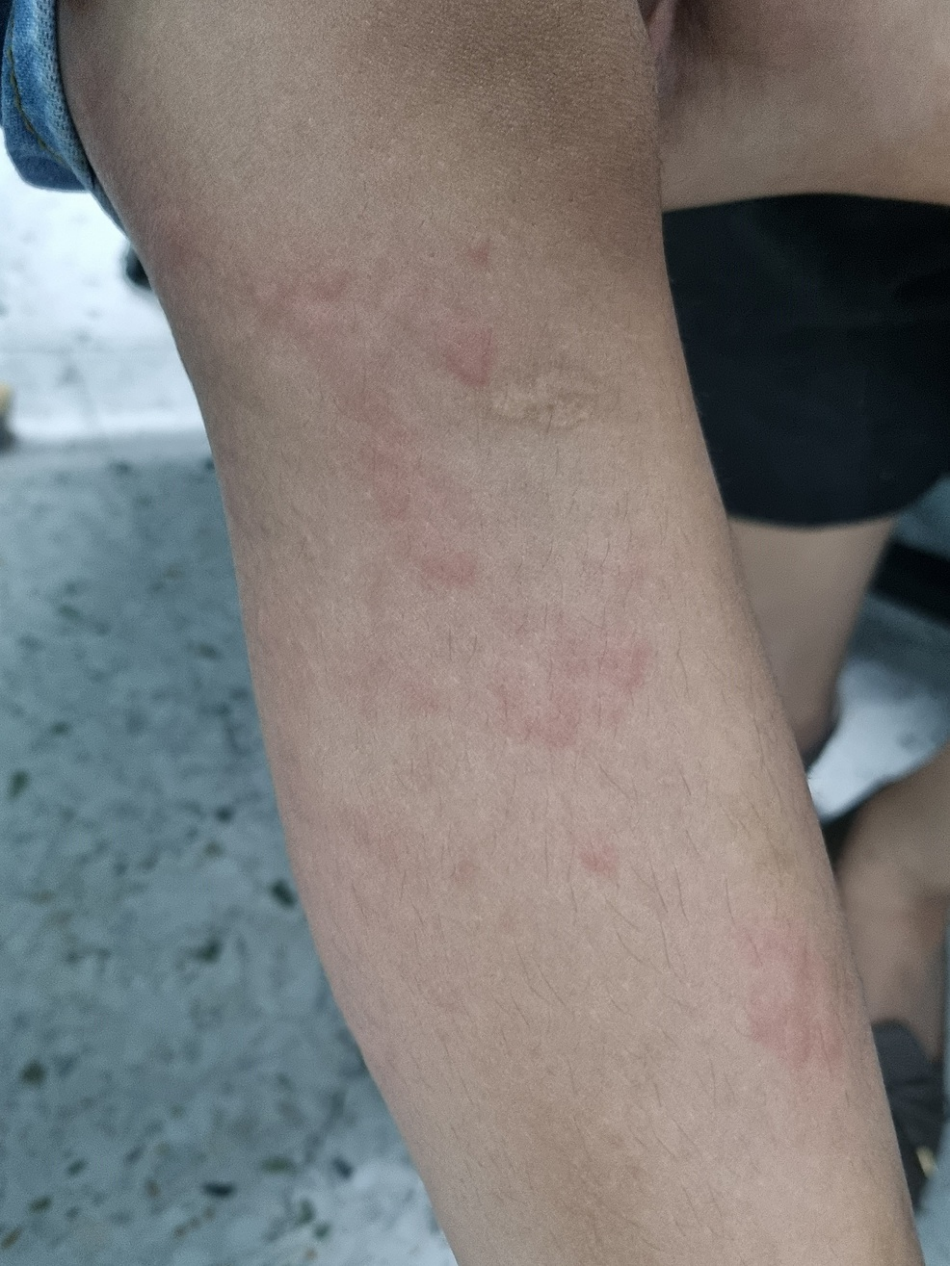
Close-up of urticaria on the child’s leg.

On examination, the child appeared alert, clinically pink, and not septic looking. Her height and weight were between the fifth to tenth percentile. Systemic examination was unremarkable. She was noted to have multiple raised areas of central swelling of various sizes with surrounding erythema distributed throughout her body. These superficial, pale-to-skin-colored swellings were consistent with wheals (Figures 1, 2).

A full blood examination showed hemoglobin of 14.0 g/dL, total white cell count of 9.1 × 10^9^/L, and platelet count of 270 × 10^9^/L. The erythrocyte sedimentation rate (ESR) was 10 mm/hour, while C-reactive protein (CRP) was 1.5 mg/L. The thyroid function test (TFT) was normal for her age.

Based on history and physical examination, the child was diagnosed with CSU. She was started on daily Loratadine of 10 mg and was reviewed two weeks later. During the review, the child reported significant improvement in her symptoms, and her parents were very much relieved.

## Discussion

Urticaria is characterized by the appearance of wheals, either with or without angioedema. It can be divided into two categories, namely, acute and chronic. The latter exhibits symptom persistence for longer than six weeks. Further classifications of chronic urticaria include inducible (occurs in reaction to identifiable triggers), or spontaneous (occurs in the absence of eliciting triggers) [1]. This discussion focuses on CSU.

Information on the prevalence of CSU in pediatric patients is very scarce. In five European nations, a study of the one-year diagnosed prevalence of CSU in pediatric patients found a prevalence of 0.75% [6]. The overall documented prevalence estimate for children is between 0.5% and 5% [1].

The autoimmune theory is the most frequently recognized approach, despite the fact that the pathophysiology of CSU is still not fully known [4]. Histamine is released when immunoglobulin (Ig)G antibodies against IgE bind to them, or when autoimmune mechanisms activate high-affinity IgE receptors on cutaneous mast cells and basophils. Both itching and swelling, which are hallmarks of urticarial lesions, are brought on by histamine [1].

In cases when CSU is suspected, the physician should aim to identify any aggravating factors, as well as should ensure that the patient does not have any evidence of a more serious illness, such as systemic lupus erythematous or urticarial vasculitis [1].

A systematic review published by Kolkhir et al. stated that CSU is strongly related to various autoimmune diseases, including Hashimoto’s thyroiditis, Grave’s disease, vitiligo, type 1 diabetes mellitus, and celiac disease. This link is more common in adults than in children [7]. A study conducted in Greece showed a low prevalence of autoimmune diseases among children with CSU [8].

The international EAACI/GA²LEN/EuroGuiDerm/APAAACI guidelines published in 2021 recommend that basic tests include differential blood counts, CRP, and/or ESR [3].

In patients with CSU, a complete blood count with differential is typically normal. When there is eosinopenia, which is defined as an absolute eosinophil count of fewer than 50 cells/µL, there is a poorer response to sgAHs and omalizumab therapy [9].

The majority of CSU cases may have normal CRP and ESR levels. Significant increases in CRP or ESR have been linked to worse outcomes, reduced quality of life, and poorer antihistamine response [10]. Such elevations ought to trigger additional investigations for systemic illnesses.

In children, CSU is rarely associated with hypothyroidism and anti-thyroid antibodies [8]. Routine measurement of TFT is not endorsed by international guidelines [5]. If thyroid disease is clinically suspected, thyroid antibodies and/or TFT should be performed [11].

The diagnosis of CSU does not routinely require a skin biopsy. However, it may be required to confirm a diagnosis of urticarial vasculitis, in lesions that last for more than 24 hours, are painful rather than itchy, or leave behind residual pigmentation [10].

The management of pediatric CSU is derived from adult guidelines, and the treatment objective is to attain complete control of the condition [1]. Therapies inhibit two pathogenic targets, namely, histamine and free IgE [12].

Guidelines have recommended a stepwise treatment approach. Due to their well-known safety profile, sgAHs are recommended as the first-line treatment in children [5]. Commonly used sgAHs in the pediatric population include loratadine, desloratadine, and cetirizine, with dosages being age-dependent [4]. If a patient shows little or no improvement after two weeks, increasing the dose up to four times is recommended [11].

The use of first-generation antihistamines in children should be avoided because they are more susceptible to sedative effects than adults are [6]. Daily administration of sgAHs is advisable to on-demand prescription because the former is more effective in preventing the development of wheals and angioedema [13].

Omalizumab, an anti-IgE, is an alternative treatment for CSU supported by strong data [9]. It prevents the activation of mast cells and basophils by inhibiting free IgEs and the interaction with their high-affinity receptor [1]. Omalizumab has been proven to be successful and safe in patients who do not demonstrate sufficient response following treatment with sgAHs [5].

Cyclosporine is an immunomodulating drug frequently used in combination with sgAHs in adults with poor symptom control [14]. It impairs the production of interleukin-4 involved in the generation of IgE, thus inhibiting IgE-mediated release of histamine [4]. Unfortunately, there is little evidence regarding the safety and usefulness of cyclosporine in pediatric patients [3].

Leukotriene receptor antagonists, in particular montelukast, have shown a reassuring safety profile in numerous studies, including in pediatric patients [4]. However, there is little proof of its effectiveness in CSU [3].

Topical steroids are not helpful in CSU. Oral glucocorticoids, on the other hand, are thought to reduce the severity of CSU due to their non-specific, broad-spectrum, anti-inflammatory properties. Well-designed studies evaluating the safety and effectiveness of glucocorticoids in pediatric CSU patients are lacking [4].

Because wheals tend to wax and wane, the weekly urticaria activity score, which is based on 24-hour self-evaluation for seven consecutive days, is a good indicator of the overall disease activity [4]. This instrument should be utilized in routine clinical practice, as it can assist in determining CSU control and response to treatment. As the severity of urticaria tends to fluctuate, and spontaneous remission can occur at any time, physicians should reevaluate the necessity for continued treatment every three to six months [5]. There is little research on the ideal length of therapy before medication tapering.

In terms of prognosis, the average duration of CSU is two to five years. The remission rates in children are observed to be a little higher than those seen in adults [1].

In adults, CSU has been shown to markedly interfere with sleep, daily activities, and work productivity [2]. The burden of disease in children has not been extensively studied. In children with CSU, their ability to learn may suffer because of time missed from school. In comparison to other childhood chronic disorders, CSU has been demonstrated to have a larger impact on health-related quality of life [6].

Unfortunately, although this condition is not uncommon in clinical practice, literature addressing the pediatric population is still lacking. Many areas of potential research remain underexplored. These include the socioeconomic consequences of pediatric CSU, as well as the usage of sgAHs for the treatment of children below six months of age, which is currently not licensed in many countries [3].

## Conclusions

This case discussion highlights the importance of identifying CSU, as the delay in obtaining the correct diagnosis may trouble the child as well as caretakers and impact the quality of life. In addition, knowledge and clarity regarding efficacious treatment options are crucial to ensure optimum control of the condition. Awareness of guidelines among physicians could lead to increased recognition of CSU and facilitate improved patient care. It is imperative that more research is conducted to fill the knowledge gaps in pediatric CSU, especially in terms of management.
